# Hyper-Pseudo-Viscoelastic Model and Parameter Identification for Describing Tensile Recovery Stress–Strain Responses of Rubber Components in TBR

**DOI:** 10.3390/polym15010076

**Published:** 2022-12-25

**Authors:** Gao Pan, Meimei Chen, Yao Wang, Jichuan Zhang, Li Liu, Liqun Zhang, Fanzhu Li

**Affiliations:** 1 State Key Laboratory of Organic-Inorganic Composites, Beijing University of Chemical Technology, Beijing 100029, China; 2 Key Laboratory of Beijing City on Preparation and Processing of Novel Polymer Materials, Beijing University of Chemical Technology, Beijing 100029, China

**Keywords:** rubber material, hyperelastic, pseudoelastic, viscoelastic, permanent set

## Abstract

Tires are often in service under dynamic conditions. Realizing the high-precision prediction of the mechanical response of rubber materials under cyclic loading can provide guidance for the design of high-performance tires. In this work, the tensile recovery stress-strain responses of rubber materials in nine different components of a truck and bus radial (TBR) tire were obtained through experiments. Before fitting, an experimental data processing method was proposed to facilitate the parameter identification for a hyper-pseudo-viscoelastic model, that is, the raw experimental data were changed to the adjusted test data. The HyperFit software was used to fit the adjusted test data based on the Yeoh hyperelastic model and the Ogden-Roxburgh pseudoelastic model to obtain the initial material parameters for the two models. In order to describe the permanent set, the Prony series viscoelastic model was introduced. The Isight software was adopted to optimize the parameters. The results showed that the hyper-pseudo-viscoelastic model (i.e., the combination of Yeoh, Ogden-Roxburgh and Prony series models) can describe the tensile recovery mechanical responses (loading curve, unloading curve and permanent set) of nine different rubber components in TBRs. The fitting results are in good agreement with the adjusted data, and all the coefficients of determination (*R*^2^) exceed 0.975. Finally, the cyclic deformation simulation of a dumbbell rubber specimen was carried out based on the above constitutive model and fitted parameters. *R*^2^ was used to describe the simulation accuracy and its value reached 0.968.

## 1. Introduction

Rubber and rubber-like materials are widely used in transportation, agriculture, medical, aviation, construction and other fields. Rubber materials play an important and irreplaceable role in cushioning, shock absorption, sealing, etc. Currently, it is reported that more than half of the world’s rubber materials are used in the automotive industry, especially in tire products. It should be pointed out that from the outside, the tire is just a round, black, unremarkable product. But in fact, tires are composed of multiple components with different functions, such as tread, sidewall, belt, shoulder wedge, carcass, bead, apex, inner liner, etc. Achieving a high-precision description of the mechanical response behavior of the rubber at these locations can provide theoretical support for the design and manufacture of high-performance tires.

The improvement of tire performance is an issue of great concern to many scholars and engineers in the rubber field. Finite element analysis (FEA) can be used as a powerful tool to analyze and study the mechanical behavior of tires. The choice of the rubber constitutive model will greatly affect the calculation accuracy and needs to be given more attention. Some outstanding works have been done in the field of structural design and performance prediction of tires. For example, some studies used the hyperelastic constitutive model to describe the large deformation behavior of the tread and sidewall, which can predict the stiffness, steady-state rolling response, contact stress, longitudinal slip and other mechanical performances of pneumatic tires [1,2,3,4]. Rugsaj and Suvanjumrat [5] applied the Ogden model to optimally design the tread and spokes of non-pneumatic tires, and proved the validity of the constitutive model and its parameters by comparing simulation data with the test results of vertical stiffness. The influence of spoke thickness on vertical stiffness was systematically analyzed. Wu et al. [6] used the combination of a hyperelastic model and pseudoelastic model to describe the hysteresis loop of the tensile recovery stress–strain curve with high fitting accuracy, and the damping performance of a smart track unit was analyzed. Ghoreishy et al. [7] introduced linear Prony series and nonlinear Bergstrom-Boyce viscoelastic constitutive models into the material models to simulate the tensile behavior of the tread component in truck and bus radial (TBR) tires. The combination of the models could accurately describe the mechanical responses under the conditions of small to medium strain and even large strain. There are also some studies that calculated the modal and rolling resistance of tires through a hyper-viscoelastic model, and the predicted results were similar to the experimental data [8,9,10]. Rosu et al. [11] considered hyper-viscoelasticity in the study of the thermal evolution of an aircraft tire rolling at high velocities up to take off values. They found that the comparison of the temperature distribution on the tire tread between numerical results and the experimental data showed the same overall trend.

Many pioneering works have been done on the performance prediction of tires using the hyper-viscoelastic constitutive model. These works laid the in-depth foundation of the research in this field. The tire is in a dynamic cyclic loading process during operation. It is necessary to describe the complex stress–strain relation of the rubber materials used in tires. When the rubber material is loaded, unloaded and then reloaded, the subsequent load required to produce the same deformation are less than the load required during the initial loading. This stress softening phenomenon is also known as the Mullins effect [12]. During cyclic loading, when the stress returns to 0, the strain is unlikely to return to 0 completely. This non-zero strain is often referred to as residual strain or permanent set.

However, to date, few studies focused on the constitutive relation of the mechanical characteristics of rubber permanent set. Two main viewpoints on the description method for the permanent set have been reported. One view is that it is due to plastic deformation, and the other view is that it is closely related to the viscous effect. Hurtado and Govindarajan [13] support the first viewpoint. They used Mises plasticity with an associated flow rule to model permanent set in the context of finite strain elasticity using a multiplicative decomposition. The stress-softening effects were modelled through a modified Ogden–Roxburgh pseudo-elastic model. Drozdov [14] derived a constitutive model for the viscoplastic response and damage of polymer composites under finite strain deformation. The model correctly described characteristic features of the Mullins effect. Unlike the standard approach, they claimed that damage serves as a measure of acceleration of plastic flow of junctions (induced by growth of free volume between chains). Huang et al. [15] found that the theory of pseudo-elasticity and the Ogden constitutive model were applicable to rubber composite, and if combined with plastic deformation, the models were more accurate for calculating the residual strain after unloading.

Some scholars support the second view. The pioneering work done by Dalrymple and Purgstaller [16] focused on the calibration of hydrogenated nitrile rubber using a Yeoh + Mullins + Prony series material model and demonstrated what is hopefully a repeatable calibration process. Fazekas and Goda [17] introduced and applied the hyper-pseudo-viscoelastic constitutive equation to simulate the complex behavior of filled rubber. The time-independent material response was described by a modified Dorfmann–Ogden pseudo-elastic material model, while the time and temperature dependences were taken into consideration by the Prony series linear viscoelastic theory and the time-temperature superposition principle. Ghoreishy and Sourki [18] selected the Arruda–Boyce model to describe the hyperelastic behavior of rubber. The parallel rheological framework (PRF) model and a new equation based on a sigmoid function were selected to describe the nonlinear viscoelastic and stress-softening effects. Kontou [19] analyzed the SBR/CNT nanocomposites exhibiting hysteresis, damage and residual strain features through a modified Gent–Zener rheological model and a damage function. Fazekas et al. [20] also proposed numerical stress solutions for predicting the stress response of hyper-visco-pseudoelastic solids. The material parameters were optimized by minimizing the difference between the predicted and the measured stress response.

**Figure 1:**
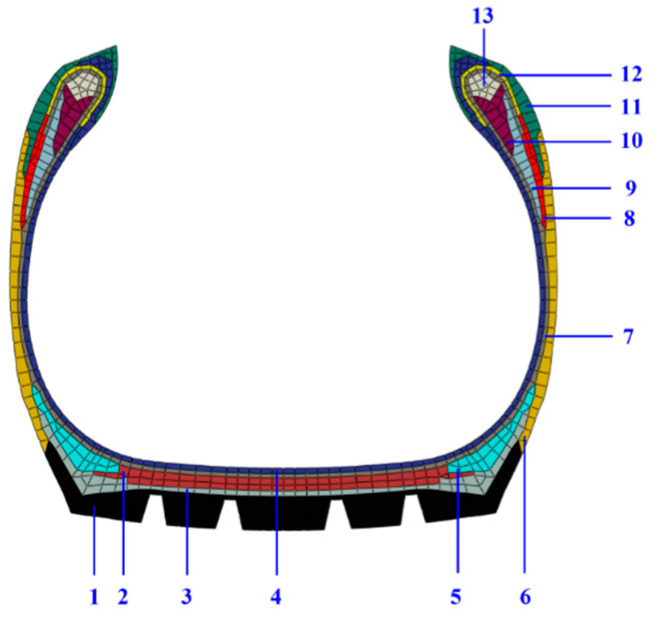
The cross-sectional profile and material distribution of the 12R22.5 TBR tire. (1) tread, (2) belt rubber, (3) under tread, (4) inner liner, (5) shoulder wedge, (6) sidewall, (7) carcass rubber, (8) filler, (9) soft apex, (10) hard apex, (11) abrasion, (12) strength, and (13) bead (steel).

However, the structure of the tire is very complex. Many rubber materials are used and the mechanical response of each material is very different, as shown in Figure 1. From the cross-sectional profile and material distribution diagram of the 12R22.5 TBR tire, it can be seen that there are 12 types of rubber components, namely tread, under tread, shoulder wedge, sidewall, carcass rubber, belt rubber, soft apex, hard apex, filler, strength, abrasion, inner liner, etc. It should be noted that the same rubber material is used in some components, like under tread and shoulder wedge, carcass rubber and belt rubber, filler and strength. In order to realize accurate simulation of tire performance, it is necessary to carry out mechanical constitutive model research on various rubber materials. Although many pioneering studies had been done to describe the finite strain elasticity, Mullins effect and permanent set of rubber material, there are few reports on accurate methods for simulating the mechanical behavior of various tire rubber materials under cyclic loading (this also makes the universality of related methods doubtful), and the related studies rarely elaborate on the detailed process of model parameter identification.

In this study, a parameter identification method is proposed for the TBR tire rubber materials (Figure 1). To present a clear picture, this article is arranged as follows: First, the workflow of how to predict the tensile recovery stress–strain responses of rubber material was expounded. Second, the cyclic loading test was carried out on nine groups of rubber materials in different components of TBR tires, and the obtained experimental data were adjusted with a novel data processing method. Next, the adjusted test data were used to identify the material parameters for the hyper-pseudo-viscoelastic model (i.e., the combination of Yeoh, Ogden-Roxburgh and Prony series model) using the parameter optimization method. Finally, the cyclic deformation simulation of a dumbbell-shaped rubber specimen was carried out based on the above constitutive model and optimized parameters. It should be pointed out that this method is not only applicable to TBR tire rubber materials, but also has some good reference value for polymer materials with similar mechanical responses.

## 2. Flow Chart of Numerical Calculation of Tensile Recovery Stress–Strain Responses

**Figure 2:**
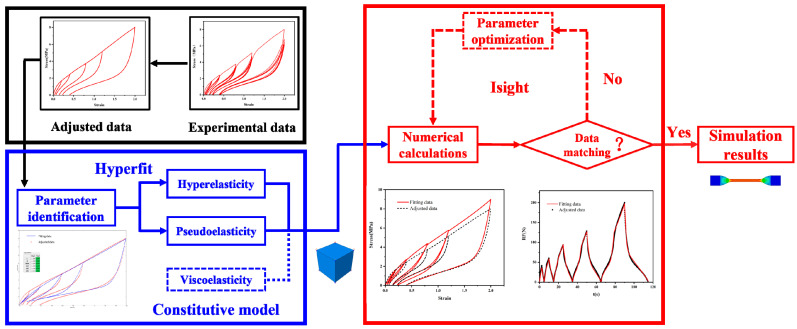
Flow chart of numerical calculation of tensile recovery stress–strain responses of rubber materials.

The flow chart of the numerical calculation of tensile recovery stress–strain responses of rubber materials is shown in Figure 2. The black box part represents the experimental data and adjustment module. The test data obtained from the quasi-static cyclic stretching experiments were processed to be adjusted data using a new method.

The blue box represents the model selection and initial parameter identification module. Based on the research work of Dalrymple [16], we also used the combination of hyperelastic + pseudoelastic + viscoelastic models to describe the mechanical responses of rubber materials under quasi-static cyclic loading. First, the material parameters of the hyperelastic and pseudoelastic models are preliminarily determined based on the adjusted data through Hyperfit. The viscoelastic constitutive model is further introduced, and the initial values of the viscoelastic material parameters are artificially set. 

The red box represents the material parameter optimization module. Under the synergy of the three constitutive models, the fitting results of the models are similar to the adjusted data in the overall trend. In order to improve the accuracy of the model, we used Isight to further optimize the material parameters. Finally, all the material parameters of the hyperelastic + pseudoelastic + viscoelastic model with better fitting effect can be obtained. These final optimized material parameters will be used for further product simulation optimization design.

## 3. Experiments and Data Adjustment

### 3.1. Material Preparation

The rubber materials were prepared by the mechanical compounding method. First, the raw rubber was masticated in an open mill (Shanghai Rubber Machinery Works No. 1, Shanghai, China) at room temperature. Zinc oxide, stearic acid and antioxidant were added in sequence and mixed. Then, carbon black was added and mixed in. Finally, the accelerator and sulfur were added and mixed to obtain the final rubber compounds. After 24 h, the rubber compounds were vulcanized in an XLB-D350 × 350 × 2-25T automatic vulcanizing press (Huzhou Dongfang Machinery Co., Ltd., Huzhou, China). The optimum cure time (t_90_) of the compounds were obtained by a P3555B2 Disc Vulcameter (Beijing Huanfeng Chemical Machinery Trial Plant, Beijing, China). The obtained vulcanized rubber was punched out by a cutter to cut out a dumbbell-shaped specimen [6].

### 3.2. Quasi-Static Cyclic Stretching Experiments

**Figure 3:**
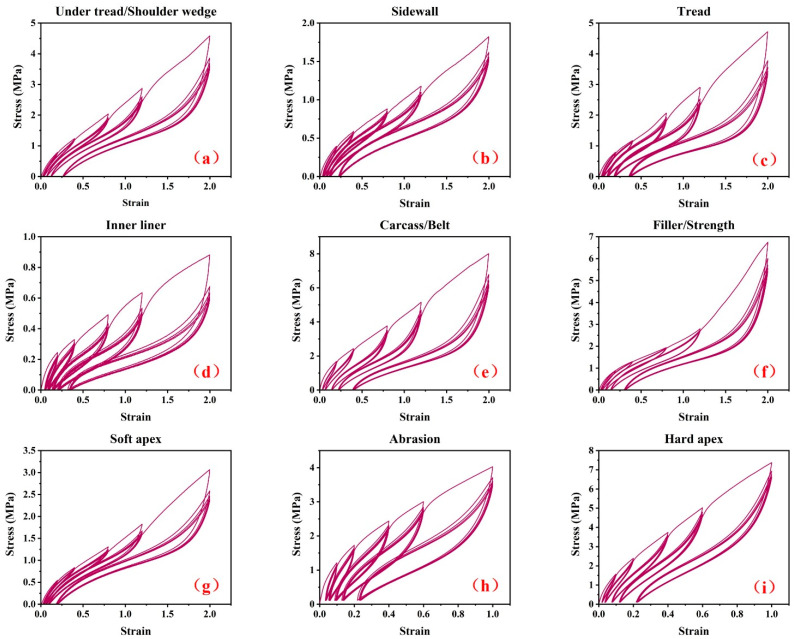
The raw stress–strain curves for nine different rubber specimens from TBR tire. (**a**) under tread and shoulder wedge, (**b**) sidewall, (**c**) tread, (**d**) inner liner, (**e**) carcass rubber and belt rubber, (**f**) filler rubber and strength rubber, (**g**) soft apex, (**h**) abrasion and (**i**) hard apex.

By using a CMT4104 electronic tensile tester (SANS, Ltd., Shanghai, China), The dumbbell-shaped specimens were stretched at a constant rate of 100 mm/min at room temperature. The quasi-static cyclic stretching experiments shown in Figure 3 were performed for nine different rubber specimens from the TBR tire. The mechanical curve in Figure 3a is for the under tread and shoulder wedge, Figure 3b is for the sidewall, Figure 3c is for the tread, Figure 3d is for the inner liner, Figure 3e is for the carcass rubber and belt rubber, Figure 3f is for the filler rubber and strength rubber, Figure 3g is for the soft apex, Figure 3h is for the abrasion, and Figure 3i is for the hard apex. The peak strain levels in Figure 3a to Figure 3g were 20%, 40%, 80%, 120% and 200%, respectively. Due to the low elongation at break for the abrasion and hard apex rubber, the peak strain levels in Figure 3h,i were 10%, 20%, 40%, 60% and 100%, respectively. Five cycles were performed for each peak strain level.

### 3.3. Experimental Data Adjustment

**Figure 4:**
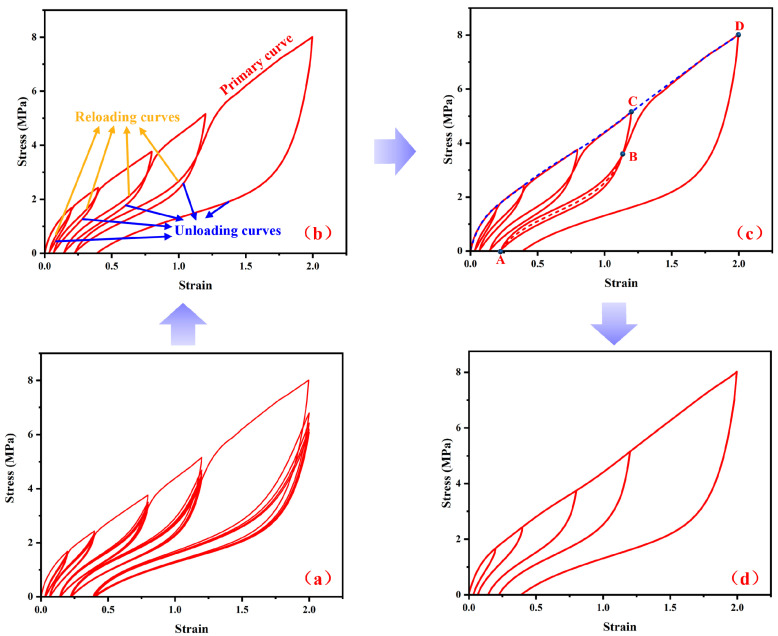
The detailed process of adjusting raw experimental data to adjusted data for the carcass rubber and belt rubber. (**a**) The raw experimental data of the carcass rubber and belt rubber, (**b**) the primary curve, unloading and reloading curves, (**c**) the redefined primary curve and intersection points A, B and C to define the adjusted unloading curves, and (**d**) the final adjusted stress strain data for parameter identification.

In this study, special attention was paid to how to accurately describe the permanent set behavior of rubber materials after tensile recovery test at different peak strain levels. The original data were adjusted to reflect the Mullins effect more intuitively. Taking the mechanical curves for the carcass rubber and belt rubber in Figure 3e as an example, the detailed adjustment process is shown in Figure 4.

Figure 4a shows the raw experimental data of the carcass rubber and belt rubber (same data as in Figure 3e). Figure 4b was obtained according to the following operations: the primary curve is kept consistent, and the curves for each unloading segment are selected as the first experimental test results, while the curves for the reloading segment at each strain level are selected as the last experimental test results. In order to explain the adjustment process more clearly, we have modified and supplemented Figure 4c on the basis of Figure 4b. Figure 4d was obtained from Figure 4c according to the following operations: the high-order polynomial was used to perform high-precision fitting and reproduction of the original test result for the primary curve, and a redefined primary curve was obtained, as shown in the blue dotted line in Figure 4c. In addition, the unloading curve corresponding to the maximum strain level remains unchanged. 

There are two intersection points between the previous unloading segment and the subsequent reloading segment. As shown in Figure 4c, there are intersection points A and B between the unloading section corresponding to the fourth strain level and the reloading section under the fifth strain level. For the data between the two points A and B, the stress adopts the mean value of reloading and unloading curves, which is shown by the red dotted line. From point B to the data point in the primary curve, we directly selected the BC curve segment. The data adjustment methods for other strain levels are also processed in this way. The final adjusted data are shown in Figure 4d.

**Figure 5:**
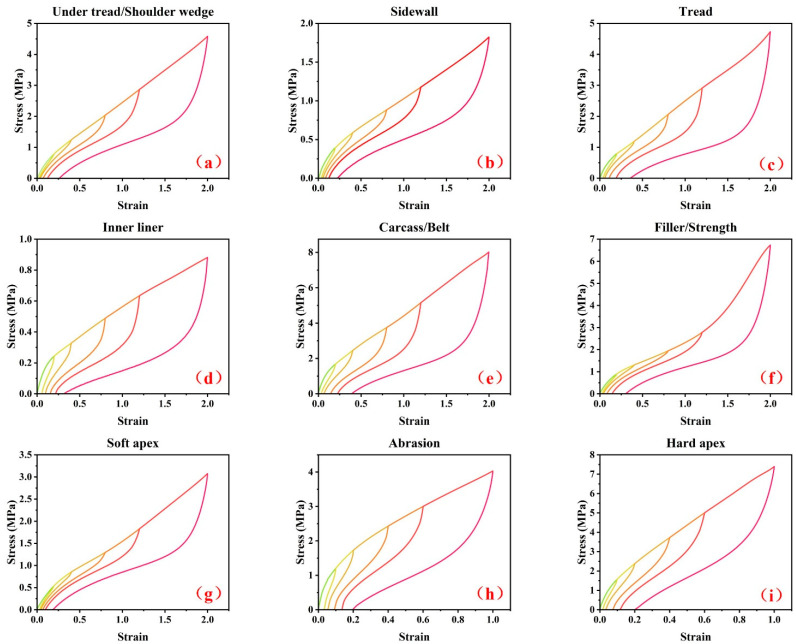
The adjusted stress–strain curves for nine different rubber specimens from TBR tire. (**a**) under tread and shoulder wedge, (**b**) sidewall, (**c**) tread, (**d**) inner liner, (**e**) carcass rubber and belt rubber, (**f**) filler rubber and strength rubber, (**g**) soft apex, (**h**) abrasion and (**i**) hard apex.

This data-processing method can ensure that the permanent sets corresponding to the original experimental data and the adjusted data do not change. Figure 5 shows the stress–strain curves of the nine rubber materials after adjustment.

## 4. Constitutive Models for Rubber Material

### 4.1. Hyperelastic Constitutive Model

The hyperelastic constitutive models of rubber and rubber-like materials have been studied for around 80 years [21]. Due to the strong nonlinearity and the nearly incompressible nature, the constitutive models of rubber are generally represented by a strain energy function (*W*) rather than a direct stress–strain relationship. The hyperelastic constitutive models can be divided into two main categories: micromechanical network models based on microstructure [22,23] and phenomenological models based on continuum mechanics [24,25,26,27]. *W* can be written in various mathematical formulas composed of strain invariants (or expressed in the form of a stretch ratio). Zhang and Li et al. [21] performed a comparative study of 85 hyperelastic constitutive models for both unfilled rubber and highly filled rubber nanocomposite materials. Some suggestions on how to select an appropriate hyperelastic constitutive model were given. In this work, the Yeoh hyperelastic constitutive model shown in Equation (1) was used for describing the primary curve. ${\bar{I}}_{1}$ is the first strain invariant and *C*_10_, *C*_20_ and *C*_30_ are three material parameters. The Yeoh model has a relatively simple form; the material constants can be obtained based on the fitting of the uniaxial tensile test data, and can reflect the inverse “S”-shaped stress–strain curve.

(1) $$W=C_{10}({\bar{I}}_{1}-3)+C_{20}{\left({\bar{I}}_{1}-3\right)}^{2}+C_{30}{\left({\bar{I}}_{1}-3\right)}^{3}$$

### 4.2. The Ogden–Roxburgh Pseudoelastic Model

In this study, the Ogden–Roxburgh pseudo-elastic constitutive model [28,29] shown in Equation (2) was used to represent the stress softening behavior of rubber. *W* is the deviatoric part of the strain energy function of the material subsequent to deformation to a given maximum degree of deformation. $\tilde{W}$ is the deviatoric strain energy function associated with primary or monotonic loading of the material prior to any preconditioning. *ϕ* is a smooth function of *η*. *η* is a damage function with a particular form shown in Equation (3). *r*, *m*, and *β* are material parameters. ${\tilde{W}}_{\max}$ is the maximum value of $\tilde{W}$ attained over the entire past history of deformation. The $\tilde{W}$ is the Yeoh model shown in Equation (1) that is used to describe the hyperelastic mechanical response. *f_er_*(*x*) shown in Equation (4) is the Gauss error function. A more detailed introduction can be found in the reference [28].

(2) $$W\left({\bar{I}}_{1},\eta \right)=\eta \tilde{W}\left({\bar{I}}_{1}\right)+\phi \left(\eta \right)$$

(3) $$\eta =1-\frac{1}{r}f_{er}\left(\frac{{\tilde{W}}_{\max}-\tilde{W}}{m+\beta {\tilde{W}}_{\max}}\right)$$

(4) $$f_{er}\left(x\right)=\frac{2}{\sqrt{\pi }}\int_{0}^{x}e^{-\omega^{2}}d\omega$$

### 4.3. Viscoelastic Constitutive Model

The research on viscoelastic constitutive models has a long history [30,31,32,33]. The linear viscoelastic model represented by the Prony series is often used to describe the viscoelastic responses (stress relaxation or creep) for traditional rubber materials. This model can express the modulus dependence of rubber material on time. The expression of shear relaxation modulus vs. time is shown in Equation (5). *G*(*t*) is the instantaneous value of the shear relaxation modulus. *G*_0_ is the initial value of the shear relaxation modulus. ${\bar{g_{i}}}^{p}$ is the dimensionless material parameter, and ${\bar{\tau_{i}}}^{p}$ is the relaxation time. *i* represents the order of the Prony series and *t* is the time. A more detailed introduction can be found in the reference [34].

(5) $$G(t)=G_{0}\left[1-\sum_{i=1}^{n}{\bar{g_{i}}}^{p}\left(1-e^{-\frac{t}{{\bar{\tau_{i}}}^{p}}}\right)\right]$$

## 5. Results and Discussion

### 5.1. Parameter Identification

**Figure 6:**
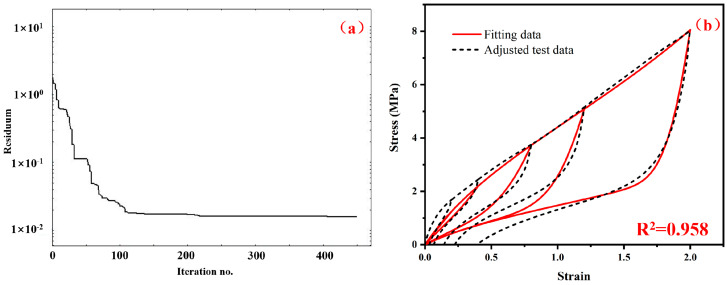
(**a**) The relation between the residuum value and the iteration number in the parameter identification process, and (**b**) comparison between the adjusted stress–strain data and the fitting results using the optimized material parameters of the hyper-pseudoelastic constitutive model.

In order to clearly introduce the process of parameter identification, the carcass rubber and belt rubber were firstly introduced as an example. The adjusted stress–strain data was processed and imported into the Hyperfit software [35]. In this study, the Yeoh hyperelastic model and the Ogden–Roxburgh pseudoelastic model were selected. The value range of material parameters in the Ogden–Roxburgh model were set as *r* > 1, *m* ≥ 0 and *β* ≥ 0. The Nelder–Mead optimization method was used for parameter identification, and the coefficient of determination *R*^2^ was used to judge the fitting accuracy. As shown in Figure 6a, when the iteration number is less than 100, the residuum value gradually decreases as the number of iterations increases. The residuum value tends to be stable as the number of iterations increases further. The optimization process of parameter identification ends when the iteration number reaches about 450. For the final fitting result shown in Figure 6b, the coefficient of determination reached 0.958.

However, it should be pointed out that the combination of hyperelastic model and pseudoelastic model cannot describe the permanent set characteristics of rubber materials. As mentioned in the introduction of this work, we intend to adopt the processing method of Dalrymple et al. [16], and introduce a viscoelastic model rather than a plastic model on the basis of the hyper-pseudoelastic constitutive model to further realize the high-precision description of the complex mechanical behavior of rubber materials. In this work, the viscoelastic model is selected as the fourth order Prony series model. The relaxation time *τ_i_* corresponding to the *i*-th order Prony series is determined empirically. Four *τ_i_* values were chosen and fixed to be 0.1, 1, 10 and 100, respectively. The initial *G_i_* values were set to be 0.2, 0.15, 0.08 and 0.05, respectively. The material parameters of the Yeoh model and Ogden–Roxburgh model were selected to be the optimized parameter results from the previous section. All the initial material parameters used in the next optimization process are shown in Table 1. These material constants serve as a good initial guesses for an optimization run.

**Figure 7:**
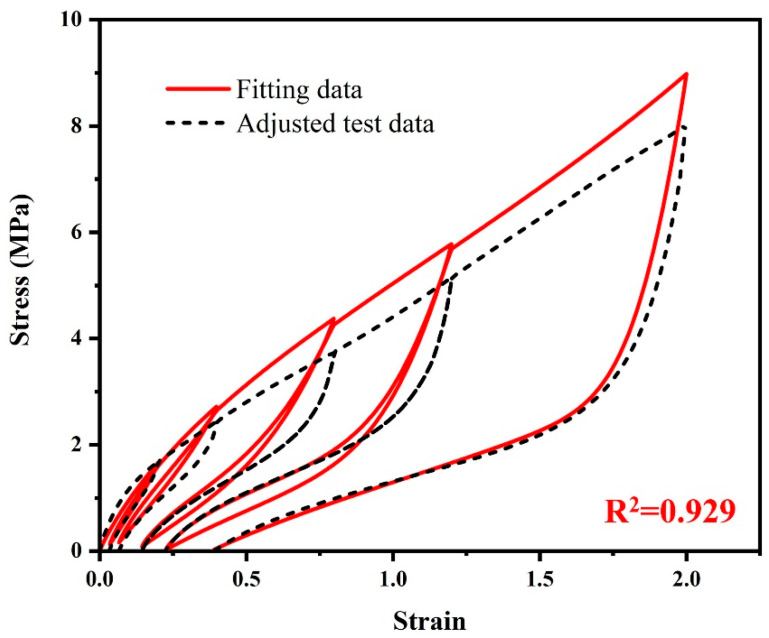
The comparison between the adjusted test data and the fitting data of the hyper-pseudo-viscoelastic model by using the initial material parameters in Table 1.

The fitting results corresponding to this set of initial material parameters for the carcass rubber and belt rubber material are shown in Figure 7. Compared with the hyper-pseudoelastic model, the permanent set appears after introducing the viscoelastic model. However, the overall loading curves shift upward as a whole, and the *R*^2^ drops from 0.958 to 0.929. Therefore, the next step is to optimize this set of material parameters. 

**Figure 8:**
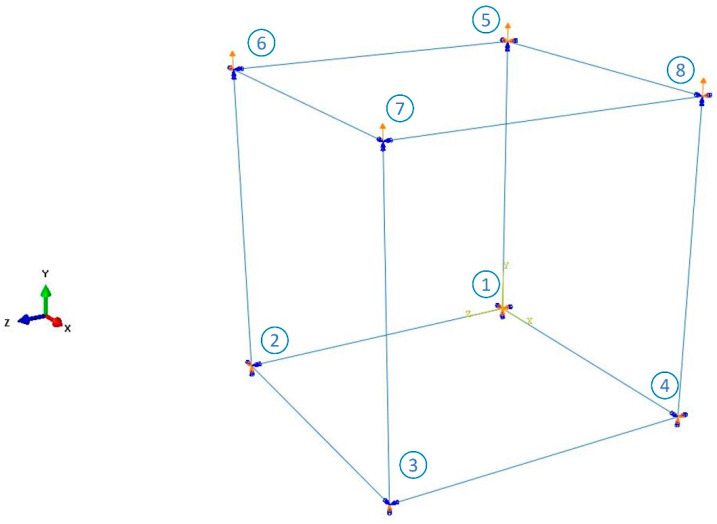
The boundary conditions for the unit cell model in the optimization process.

The Isight software was adopted to optimize the material parameters of the hyper-pseudo-viscoelastic model (i.e., the combination of Yeoh, Ogden–Roxburgh and Prony series models) that can describe the tensile recovery mechanical responses (loading curve, unloading curve and permanent set) of rubber. In order to improve the computational efficiency of optimization, an 8-node unit cell model (C3D8RH) was established. The model size is 10 mm × 10 mm × 10 mm. Tensile recovery displacements in the vertical direction acted on nodes 5, 6, 7 and 8. The other four nodes 1, 2, 3 and 4 were fixed in the simulation as shown in Figure 8. After calculation, the reaction force (RF2) vs. displacement (U2) curve for the unit cell was obtained. Then the force vs. displacement data was postprocessed to be stress vs. strain data. 

**Figure 9:**
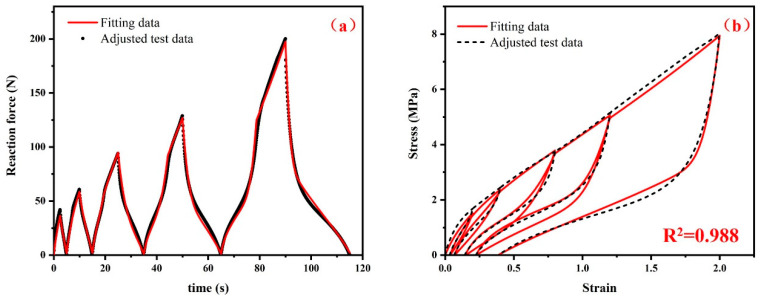
(**a**) The relationship between the reaction force and time when the sum of squared difference reaches a minimum value in the parameter identification process, and (**b**) comparison between the adjusted stress–strain data and the fitting results using the final optimized material parameters of the hyper-pseudo-viscoelastic constitutive model.

In the optimization process, the Hooke–Jeeves direct search method as a robust local optimization algorithm was utilized. The Hooke–Jeeves technique begins with a starting guess and searches for a local minimum. Since the approximate range of parameters has been determined during parameter identification in the early stage, the problem that the optimization method is prone to fall into a local optimal solution can be avoided as much as possible. The number of optimizations was set to 600. This setup balances computational efficiency with adequate optimization calculations. The material parameters were iterated until the sum of the squared difference between adjusted data and calculation data of the RF (reaction force)-t (time) curve reaches a minimum value. Figure 9a shows the relation between the reaction force and time when the sum of the squared difference reaches a minimum value. The optimized material parameters are recorded in Table 2. 

The comparison between the adjusted stress–strain data and the fitting results using the final optimized material parameters of the hyper-pseudo-viscoelastic constitutive model is shown in Figure 9b. The permanent set was described with high accuracy. The relative errors of the permanent set between the adjusted test data and the fitting data at different peak strain levels are recorded in Table 3. Except for the first strain level, the relative errors between the adjusted test data and the fitting data of permanent set at other strain levels are all less than 5%.

Before optimization, the calculated results were close to the adjusted data at small strains, while deviations occurred at medium and large strains as shown in Figure 7. After optimization, there was a certain difference between the adjusted data and fitting data for the loading curve at small strains from 0 to 40% as shown in Figure 9b. This is due to the balance made to improve the fitting results under the combined action of hyperelastic, viscoelastic and pseudoelastic constitutive models. However, the overall fitting effect has been greatly improved, and the coefficient of determination has increased from 0.929 to 0.988.

**Figure 10:**
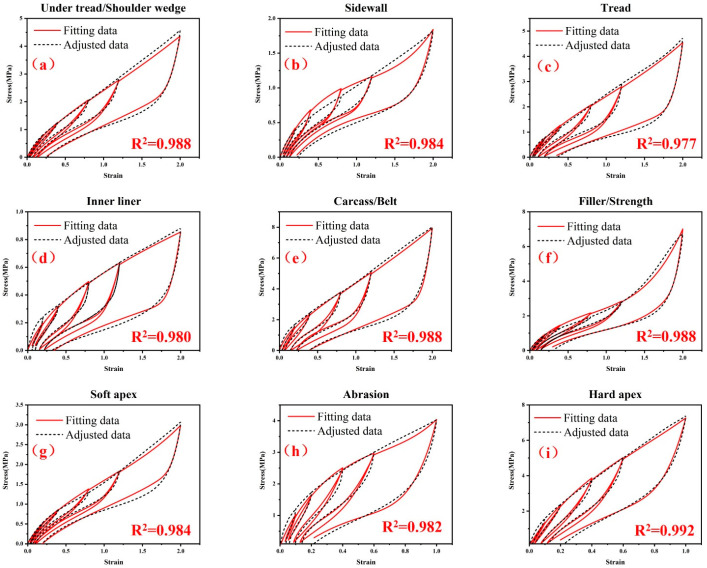
Comparison between the adjusted stress–strain data and the fitting results using the final optimized material parameters of the hyper-pseudo-viscoelastic constitutive model for the nine rubber materials in TBR tires: (**a**) under tread and shoulder wedge, (**b**) sidewall, (**c**) tread, (**d**) inner liner, (**e**) carcass rubber and belt rubber, (**f**) filler rubber and strength rubber, (**g**) soft apex, (**h**) abrasion and (**i**) hard apex.

For materials used in other rubber components in TBR, the same material parameter identification method was utilized. In order to make the description of the method not too verbose, only the final comparison results of the other eight rubber materials are shown Figure 10. The tensile recovery stress–strain responses of all the nine rubber materials can be well described. The coefficients of determination *R*^2^ are all great than 0.975. The lowest value is 0.977, and the highest is 0.992. The *R*^2^ for the under tread and shoulder wedge is 0.988. The *R*^2^ for the sidewall is 0.984. The *R*^2^ for the tread rubber is 0.977. The *R*^2^ for the inner liner is 0.980. The *R*^2^ for the filler and strength rubber is 0.988. The *R*^2^ for the soft apex is 0.984. The *R*^2^ for the abrasion rubber is 0.982, and the *R*^2^ for the hard apex is 0.992. 

### 5.2. Finite Element Analysis of the Tensile Recovery of Dumbbell-Shaped Specimen

After completing the identification of the material parameters in the hyper-pseudo-viscoelastic constitutive model of rubber materials, the next important point is to verify the validity of the model in the finite element analysis. We used the tensile recovery deformation of a dumbbell-shaped rubber specimen as the analysis object.

**Figure 11:**
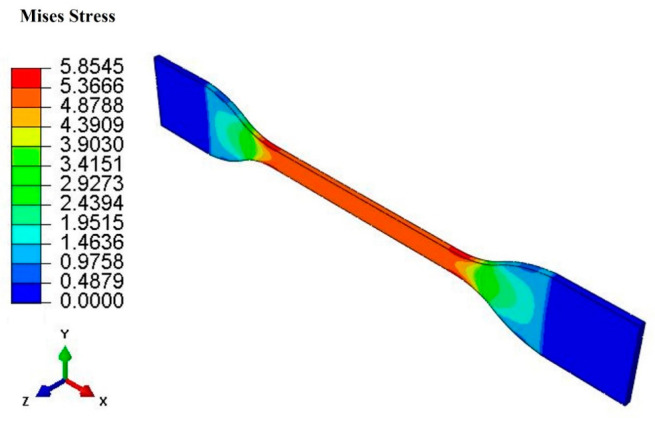
Contour plot of the Mises stress obtained at the peak strain of 40% for the dumbbell-shaped specimen.

In the finite element model, the thickness of the three-dimensional dumbbell-shaped rubber specimen is set to 1 mm. The width and length of the middle working area is 4 mm and 25 mm, respectively. The model is composed of 13,068 elements. The element type of the model was an 8-node linear hybrid, constant pressure and reduced integration brick element with enhanced hourglass control (i.e., C3D8RH in Abaqus nomenclature). Tensile recovery of the hard apex dumbbell-shaped specimen with three different peak strain levels (10%, 20%, and 40%) was simulated. The contour plot of the Mises stress obtained at the peak strain of 40% is shown in Figure 11.

**Figure 12:**
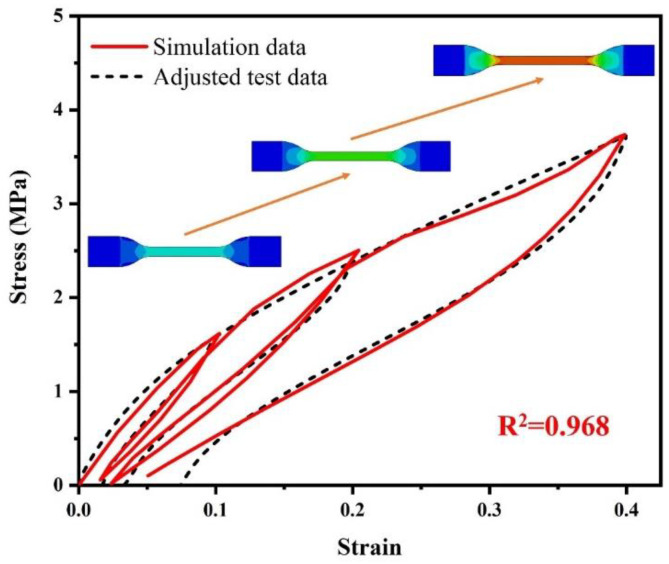
The comparison between the simulation data and adjusted test data of tensile recovery mechanical response of hard apex dumbbell-shaped rubber specimen.

Figure 12 shows the comparison between the simulation data and adjusted test data of tensile recovery mechanical response of the hard apex dumbbell-shaped rubber specimen. The coefficient of determination *R*^2^ reaches 0.968. Except for the deviation of the permanent set at high peak strain levels, the overall description of the hyper-pseudo-viscoelastic constitutive model performs well.

## 6. Conclusions

1. Nine rubber materials used in different components of TBR tires were subjected to tensile recovery experiments with different peak strain levels. An experimental data processing method was proposed to facilitate the parameter identification for the hyper-pseudo-viscoelastic model. The workflow of how to perform numerical calculation of tensile recovery stress–strain responses of rubber materials in tires was described.
2. The Yeoh hyperelastic model, the Ogden–Roxburgh pseudoelastic model and the Prony series viscoelastic model were adopted together to describe the tensile recovery mechanical responses (loading curve, unloading curve and permanent set) of nine different rubber materials. The fitting result data are all in good agreement with the adjusted test data, and all the coefficients of determination exceeded 0.975. This method has certain universality.
3. This study indicates that using the hyper-pseudo-viscoelastic constitutive model to predict the quasi-static cyclic loading of rubber materials is a feasible method. This work can guide the mechanical research of soft substances and rubber-like materials, and provides a support to design high-durability rubber products and high-performance tires.
4. However, the introduction of complex nonlinear constitutive equations usually makes the finite element analysis have great convergence problems. In this work, the hyper-pseudo-viscoelastic constitutive model is only used for the deformation analysis of a simple dumbbell-shaped rubber specimen. For more complex products such as tires, the applicability of these models needs to be further explored.

## Figures and Tables

**Table 1:** The initial material parameters in hyper-pseudo-viscoelastic model for the carcass rubber and belt rubber.

Yeoh Hyperelastic Model		Ogden-Roxburgh Pseudoelastic Model		Prony Series Viscoelastic Model			
*C*10	1.2286	*r*	1.5022	*G*_1_	0.20	*τ*_1_	0.1
				*G*_2_	0.15	*τ*_2_	1.0
*C*20	0.0055	*m*	1.3038				
				*G*_3_	0.08	*τ*_3_	10.0
*C*30	0.0007	*β*	0.0605				
				*G*_4_	0.05	*τ*_4_	100.0

**Table 2:** The optimized material parameters in hyper-pseudo-viscoelastic model for the carcass rubber and belt rubber.

Yeoh Hyperelastic Model		Ogden–Roxburgh Pseudoelastic Model		Prony Series Viscoelastic Model			
*C*10	0.9750	*r*	1.7874	*G*_1_	0.0303	*τ*_1_	0.1
				*G*_2_	0.3588	*τ*_2_	1.0
*C*20	0.0156	*m*	0.7440				
				*G*_3_	0.0742	*τ*_3_	10.0
*C*30	0.0001	*β*	0.0331				
				*G*_4_	0.0641	*τ*_4_	100.0

**Table 3:** The relative errors of permanent set between the adjusted test data and the fitting data at different peak strain levels for the carcass rubber and belt rubber.

The Peak Strain Level	Permanent Set of the Adjusted Test Data	Permanent Set of the Fitting Data	Relative Error
20%	0.033	0.037	12.1%
40%	0.066	0.069	4.5%
80%	0.143	0.150	4.9%
120%	0.224	0.231	3.1%
200%	0.387	0.392	1.3%

## Data Availability

The data presented in this study are available on request from the corresponding author. The data are not publicly available due to privacy.
